# Authentication of Edible Insects’ Powders by the Combination of DART-HRMS Signatures: The First Application of Ambient Mass Spectrometry to Screening of Novel Food

**DOI:** 10.3390/foods11152264

**Published:** 2022-07-29

**Authors:** Alessandra Tata, Andrea Massaro, Filippo Marzoli, Brunella Miano, Marco Bragolusi, Roberto Piro, Simone Belluco

**Affiliations:** 1 Laboratorio di Chimica Sperimentale, Istituto Zooprofilattico Sperimentale delle Venezie, 36100 Vicenza, Italy; amassaro@izsvenezie.it (A.M.); bmiano@arpae.it (B.M.); mbragolusi@izsvenezie.it (M.B.); rpiro@izsvenezie.it (R.P.); 2 Department of Food Safety, Istituto Zooprofilattico Sperimentale delle Venezie, 35020 Legnaro, Italy; fmarzoli@izsvenezie.it (F.M.); sbelluco@izsvenezie.it (S.B.)

**Keywords:** data fusion, frauds, fingerprinting, house cricket, yellow mealworm, migratory locust, silk moth, DART-HRMS

## Abstract

This feasibility study reports the use of direct analysis in real-time high-resolution mass spectrometry (DART-HRMS) in profiling the powders from edible insects, as well as the potential for the identification of different insect species by classification modeling. The basis of this study is the revolution that has occurred in the field of analytical chemistry, with the improved capability of ambient mass spectrometry to authenticate food matrices. In this study, we applied DART-HRMS, coupled with mid-level data fusion and a learning method, to discriminate between *Acheta domesticus* (house cricket), *Tenebrio molitor* (yellow mealworm), *Locusta migratoria* (migratory locust), and *Bombyx mori* (silk moth). A distinct metabolic fingerprint was observed for each edible insect species, while the *Bombyx mori* fingerprint was characterized by highly abundant linolenic acid and quinic acid; palmitic and oleic acids are the statistically predominant fatty acids in black soldier fly (*Hermetia illucens*). Our chemometrics also revealed that the amino acid proline is a discriminant molecule in *Tenebrio molitor*, whereas palmitic and linoleic acids are the most informative molecular features of the house cricket (*Acheta domesticus*). Good separation between the four different insect species was achieved, and cross-validation gave 100% correct identification for all training samples. The performance of the random forest classifier was examined on a test set and produced excellent results, in terms of overall accuracy, sensitivity, and specificity. These results demonstrate the reliability of the DART-HRMS as a screening method in a future quality control scenario to detect complete substitution of insect powders.

## 1. Introduction

Insects employed as ingredients, food, and/or feed are arousing interest worldwide. More than 1900 insect species have reportedly been used as food in the world, especially in developing countries [1]. The high environmental sustainability, with the efficient use of land and water resources and decreased greenhouse gas emissions, are some of the advantages of insect farming, in comparison to intensive livestock breeding [2]. Moreover, insects are a valuable nutritive source of high-value proteins, fats, minerals, vitamins, and fiber (this last is due to the presence of chitin) [3].

The European community categorizes insect-based food as novel food according to Reg. 2015/2283 [4]. This legislation regulates the production and marketing of insects as food in Europe. In the first European Food Safety Authority (EFSA) opinion on the risk profiles of edible insects, which followed previous opinions of national food safety authorities, a starting list of 12 species of insects was considered, including the silkworm *Bombyx mori* and black soldier fly *Hermetia illucens* [5]. Based on the positive scientific opinion of EFSA, the European Commission has already authorized the trade of some processed insects (to date, the house cricket *Acheta domesticus*, yellow mealworm *Tenebrio molitor*, and migratory locust *Locusta migratoria*), as well as some other species, are under evaluation. Nowadays, Regulation (EU) No. 2017/2470 allows the trade of insect-based products, either whole or in the form of a powder [6], according to the applicant request. For this latter form, the risks associated with food fraud by replacing the declared species with less valuable one(s) must be taken into account. FAO advised that edible insects as powder or flour could be purchased by consumers and used as an ingredient in baked goods [1]. For this reason, in 2021, FAO recommended the development of analytical methods to verify the authenticity of insect powders, as mislabeling will impact consumer confidence and expose individuals to allergy risks [7]. Today, ingredients of animal origin are primarily authenticated by using polymerase chain reaction (PCR), following the provisions described in Annex VI of EU Commission Regulation No. 2019/152 [8]. To the best of our knowledge, only a few studies have been published regarding the authentication of insect species by chemical fingerprinting. Non-targeted proteomics were applied to determine insect species-specific marker peptides in highly processed insect meal from five different species by liquid chromatography–high-resolution mass spectrometry, in order to allow for species identification [9]. Liquid chromatography mass spectrometry was recently applied to investigate and characterize the lipids of *Tenebrio molitor* and the nutritional composition of eight terricolous insects [10,11]. Infrared spectroscopy (IR), using attenuated total reflectance mid-infrared spectroscopy, combined with multivariate analysis, has been extensively used in rapid chemical fingerprinting of edible insect powders [12]. Edible insects were also analyzed by matrix-assisted laser desorption ionization–time of flight mass spectrometry (MALDI–TOF MS), thus enabling the precise identification of the different species [13].

Direct analysis in real-time mass spectrometry (DART-MS) is one of the most common mass spectrometric “ambient ionization” sources and works at ambient conditions, without prior chromatographic separation. Specifically, DART is characterized by a heated plasma discharge of helium that reacts with the atmospheric molecules (N_2_, H_2_O, and O_2_). The radical atmospheric molecules interact with the analytes of samples, resulting in the ionization and desorption of the latter [14]. Several studies demonstrated its successful application for animals [15], vegetables [16], and bacterial species identification [17,18]. Necrophagous insects of forensic importance were recently classified by chemical fingerprint signatures acquired by DART-HRMS [19]. In this feasibility study, we applied DART-HRMS, coupled with mid-level data fusion and learning methods, for the purpose of discriminating powdered *Acheta domesticus*, *Bombyx mori*, *Hermetia illucens*, and *Tenebrio molitor*.

## 2. Materials and Methods

### 2.1. Samples

Twelve samples of raw powdered *Acheta domesticus* adults bred in Italy or Thailand, 11 samples of raw powdered *Bombyx mori* pupae farmed on six different Italian farms, 5 samples of raw powdered *Hermetia illucens* larvae produced in Italy, and 5 samples of powdered *Tenebrio molitor* larvae bred in Italy, France (cooked and dried, purchased from retail), or Switzerland (purchased from retail) were studied. The analyzed insect life stages of *A. domesticus* and *T. molitor* are those approved for retail in Europe (Regulation (EU) 2017/2470) [6]. 

### 2.2. Sample Preparation

Two different extraction procedures were applied to the edible insect powders to achieve a more comprehensive exploration of the chemical changes among edible insects’ powders. While selectivity and optimization and recovery validation of the extraction procedures is desirable in targeted methods, fingerprinting approaches require a non-selective sample preparation to detect a broad range of substances in the sample [20]. For this reason, in the first extraction, 0.5 g of sample were diluted in 10 mL of a solution of water and methanol (H_2_O:MeOH; 20:80 *v*/*v*) (MilliQ water and methanol HPLC-grade with 99.9% purity, from Sigma Aldrich, St. Louis, MI, USA), vortexed for 30 s, sonicated for 15 min, and centrifuged for 5 min at 12,000× *g*. We will name this type of extraction in the text: “extraction A”. In the second protocol, 0.5 g of sample were suspended in 10 mL of ethyl acetate (EtAc) (99.9% purity, Sigma Aldrich, St. Louis, MI, USA), vortexed for 30 s, and then sonicated for 15 min. A 1 mL volume of this extract was transferred into a small tube and centrifuged for 5 min at 12,000× *g*. We will name this extraction: “extraction B”.

### 2.3. DART-HRMS

A DART SVP 100 ion source (IonSense, Saugus, MA, USA), coupled with an Exactive Orbitrap (Thermo Fisher Scientific, Waltham, MA, USA), was used. Five mL of each extract were placed on a glass capillary rod. A Dip-it(R) autosampler automatically positioned the glass capillary rod in front of the source (IonSense, Saugus, MA, USA). The DART parameters were optimized as follows: grid voltage 100 V; helium flow 4.26 L/min; temperature 350 °C; and single run time 0.66 min. The DART source was coupled to an autosampler that transfers the sample, pipetted on a capillary rod, between the gun source, and the MS inlet with a speed of 0.3 mm/s. Mass spectrometer settings were as follows: S-lens RF level, 55; capillary temperature, 250 °C; and maximum injection time, 10 ms.

The resolution was set to 70,000 full width at half maximum (FWHM), and the mass spectra were acquired in the range of 75–1125 Da in positive and negative ion modes. Note that each extract (A and B) was analyzed both in positive and negative ion mode in triplicate.

The DART-HRMS spectra were opened and visualized by using XCalibur QualBrowser software (Thermo Fisher Scientific, Waltham, MA, USA) These were converted to mzML files using Proteowizard and then converted into.csv files with Rstudio 3.6.1 software (RStudio Team, 2016; RStudio Integrated Development for R; RStudio, Inc., Boston, MA, USA). The tentative assignment of the ions was done by interrogating the human metabolome database (HMDB, www.hmdb.ca, accessed on 18 July 2021) library. In order to confirm an ion assignment retrieved by the HMDB library, a literature search was also carried out. The literature, reporting high levels or previous observations of the assigned molecules, helped to confirm their presence in the spectra.

### 2.4. Data Fusion and Statistical Analysis

The triplicate spectral data were statistically analyzed using MetaboAnalyst 5.0 web portal (www.metaboanalyst.ca, accessed on 24 July 2021) and Rstudio 3.6.1 software. The isotopes were removed, and the *m*/*z* values aligned. The the *m*/*z* with more than 75% of missing ion intensity were removed over all the data. The ions with <75% of missing values were each replaced with the value of 1/5 of the lowest acquired intensity. The signals were normalized by sum, whereas each feature was normalized by Pareto scaling. For an initial exploration, the data were concatenated by low-level data fusion [21,22,23] (Massaro, 2021, *New strategies for the differentiation of fresh and frozen/thawed fish: A rapid and accurate non-targeted method by ambient mass spectrometry and data fusion* (part A); Tata, 2022, *Ambient mass spectrometry for rapid authentication of milk from Alpine or lowland forage*; Tata, 2022, *Detection of soft-refined oils in extra virgin olive oil using data fusion approaches for LC-MS, GC-IMS, and FGC-Enose techniques: The winning synergy of GC-IMS and FGC-Enose*) and submitted to principal component analysis (PCA). Afterwards, the data were split into training (25 samples) and test (8 samples) sets; the training set was used to build the classification model, and the test set was withheld for further validation of the model. The mid-level data fusion approach followed that described by Massaro et al. [24]. Briefly, each normalized training set was submitted to supervised partial least squared discriminant analysis (PLS-DA). The first five PLS-DA score components of each data block were used to retrieve the most informative variables. The 18 selected informative variables were submitted to hierarchical cluster analysis with Pearson distance and Ward linkage to investigate the correlation between the four insect species and ionic features.

Heatmaps, graphical representations of the informative molecular features retrieved by mid-level data fusion, were constructed where each row represents a different ion (*m*/*z* value), and the columns are the insect species. The intensity (red-blue color bar) directly correlates to the relative intensity of that ion in the spectrum of each insect.

### 2.5. Creation of the Classifier and Its Validation

A random forest classification model was constructed with the 18 selected informative ions, and the model’s ability to correctly classify the samples (in triplicate) in the training set was verified. A 10-times repeated 5-fold cross-validation was performed. The performance of the random forest model was evaluated on the test set withheld previously The performance of a classifier were expressed, in terms of true positive rate (sensitivity), true negative rate (specificity), and accuracy. The accuracy is the number of correct predictions divided by the total number of predictions. The true positive rate (sensitivity) of a classifier is defined as: True positive rate = True positives/(True positives + False negatives). On the other hand, the true negative (specificity) rate is calculated as: True negative rate = True negatives/(True negatives + False positives). The number of true positive, true negative, false positive and false negative are derived from the confusion matrices.

## 3. Results

**Figure 1:**
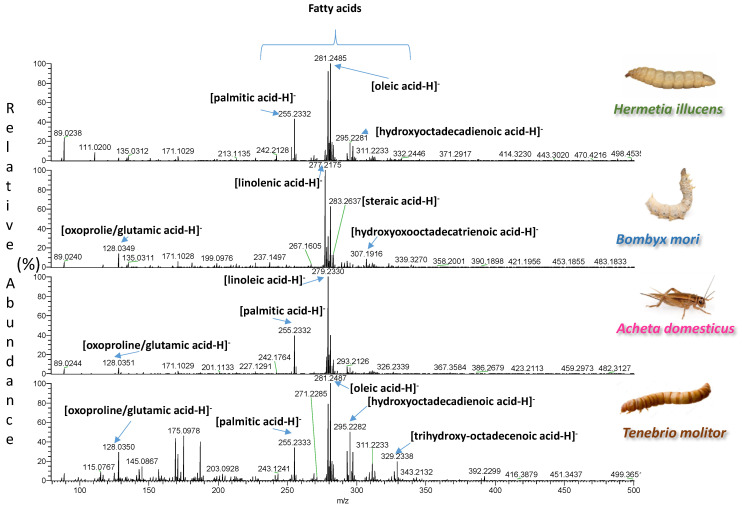
DART-HRMS spectra of the extracts B of edible powders of *Hermetia illucens*, *Bombyx mori*, *Acheta domesticus*, and *Tenebrio molitor* acquired in negative ion mode. The acquired spectra are zoomed in, to facilitate the visualization of the most informative *m*/*z* range between 75–500 Da.

**Figure 2:**
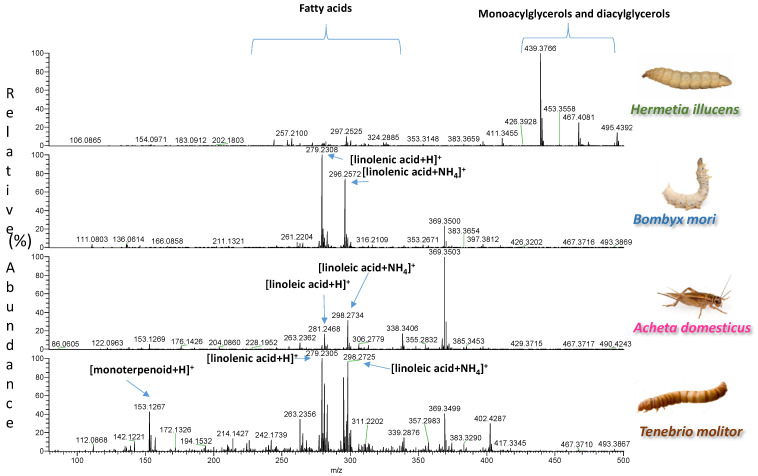
DART-HRMS spectra of the extracts B of edible powders of *Hermetia illucens*, *Bombyx mori*, *Acheta domesticus*, and *Tenebrio molitor* acquired in positive ion mode. The acquired spectra are zoomed in, to facilitate the visualization of the most informative *m*/*z* range between 75–500 Da.

**Figure 3:**
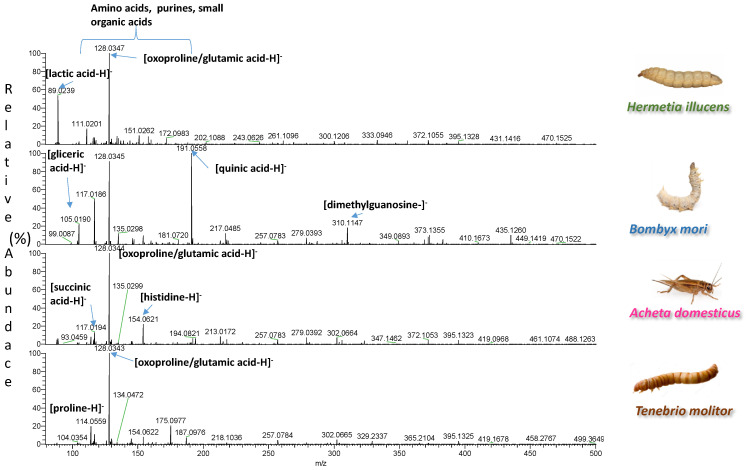
DART-HRMS spectra of the extracts A of edible powders of *Hermetia illucens*, *Bombyx mori*, *Acheta domesticus*, and *Tenebrio molitor* acquired in negative ion mode. The acquired spectra are zoomed in, to facilitate the visualization of the most informative *m*/*z* range between 75–500 Da.

**Figure 4:**
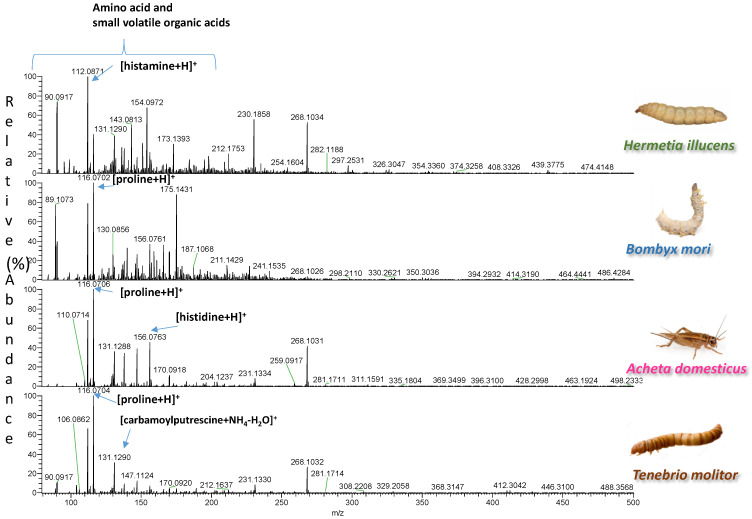
DART-HRMS spectra of the extracts A of edible powders of *Hermetia illucens*, *Bombyx mori*, *Acheta domesticus*, and *Tenebrio molitor* acquired in positive ion mode. The acquired spectra are zoomed in, to facilitate the visualization of the most informative *m*/*z* range between 75–500 Da.

A total of 33 samples, representing four different insect species, were extracted with two types of solvents. The spectra of the two types of extracts were easily acquired by DART-HRMS in positive and negative ion mode under soft ionization conditions. Each analysis was accomplished in less than 0.6 min. The blank spectra of the extraction solvents are reported in Figures S2–S5. Representative spectra illustrating signals from one sample per species are shown in Figure 1, Figure 2, Figure 3 and Figure 4. In each spectrum, characteristic signals are highlighted. The observed compounds were consistent with those that are expected to be present in dried powders rich in proteins and fat. The extracts B of insects with fingerprints acquired in negative ion mode were characterized by deprotonated saturated and unsaturated fatty acids (Figure 1 and Figure 2). Amino acids, oxidized amino acids, and aldehydes were observed in the extracts A of all insects when fingerprints were acquired in negative ion mode (Figure 3 and Figure 4).

PCA was applied to data merged by low level data fusion to visualize the molecular variation between insects’ edible powders. From the two-dimensional representation of the first two principal components (PC1 and PC2) scores, a tendency for clustering can be observed (Figure S1). Afterwards, each separate species’ fingerprints were merged by mid-level data fusion, and the retrieved markers processed by multivariate statistical analysis methods to enable rapid species-level classification.

**Figure 5:**
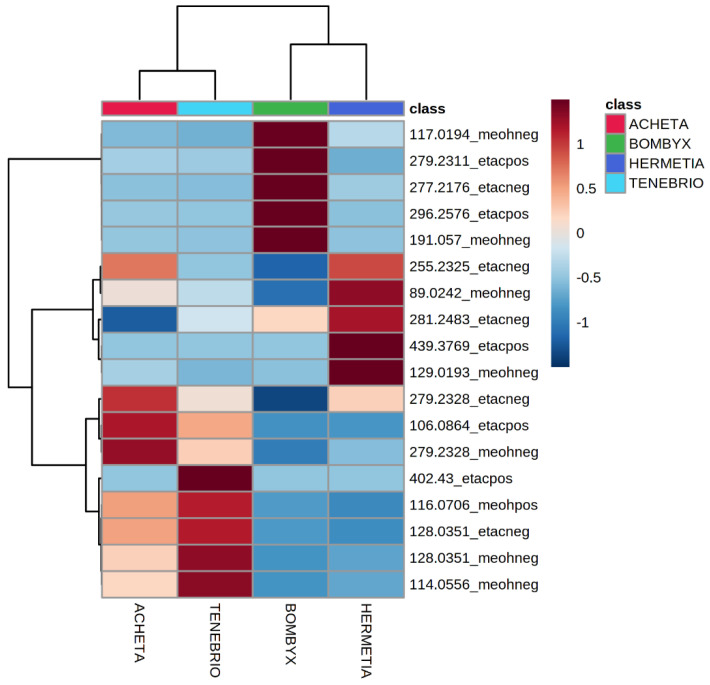
Heatmap (Pearson distance, Ward clustering algorithm) shows the correlation between selected informative variables (*m*/*z*) and different insect powders. The acronym “etacpos” indicates that the informative variable was retrieved from the dataset obtained by the analysis of the extract B in positive ion mode; the acronym “etacneg” indicates that the ion was retrieved from the extract B analyzed by negative ion mode; the acronym “meohpos” indicates that the informative ion was extrapolated from the dataset of the extract A analyzed in positive ion mode; the acronym “meohneg” indicates that the informative ion was selected from the dataset obtained by the analysis of the extract A in negative ion mode.

The heatmap confirmed that the mass spectra profiles vary between insect species (Figure 5). The tentative assignments of the informative molecular features in the merged fingerprints for each species are listed in Table 1. The literature that aided their assignment is listed in the last column on the right.

In detail, *Tenebrio molitor* powders were characterized by a high relative abundance of protonated and deprotonated proline, as well as deprotonated glutamic acid. Ammoniated butyric acid, deprotonated palmitic acid, and linoleic acid were the compounds that contributed the most to the identification of *Acheta domesticus*. *Bombyx mori* powders were differentiated by highly abundant polyunsaturated linolenic acid and deprotonated quinic acid (Table 1). Deprotonated lactic acid, palmitic acid, oleic acid, and monoacylglycerol MAG (24:1) characterized the *Hermetia illucens* powders (Table 1).

The molecular features of the heatmap were used to build up a random forest classifier. Good separation between the four different insect species was observed, and cross-validation gave 100% correct identification for all training samples (Table 2).

The performance of the random forest classifier was evaluated on the test set and achieving excellent results, in terms of overall accuracy, sensitivity, and specificity (Table 2). Confusion matrices with the results of the validation are reported in the supporting info (Tables S1 and S2). 

## 4. Discussion

In this feasibility study, we first retrieved the chemical biomarkers of each insect species by mid-level data fusion and then used these molecular features to build a classification model for the authentication of unknown samples. Although no full identification of the informative molecules was performed by MS/MS fragmentation, the biomarkers were putatively assigned by HMDB library and bibliography searches. As shown in Table 1, the *Bombyx mori* fingerprint was characterized by highly abundant linolenic and quinic acids. While the presence of quinic acid is due to the insect’s mulberry-based diet, linolenic acid is known to be the predominant fatty acid in oil obtained from desilked silkworm pupae [26]. In accordance with the literature that proved the major fatty acids in *Acheta domesticus* are linoleic (30–40%) and palmitic (24–30%) acids [25], these same lipids were the most discriminant biomarkers in the DART-HRMS *Acheta domesticus* fingerprint. In the same vein, palmitic and oleic acids are the predominant fatty acids in black soldier fly (*Hermetia illucens*) [27,28], and they were the most informative molecular features of our DART-HRMS-based classifier. Our chemometrics revealed that the amino acid proline (*m*/*z* 114.0556 and *m*/*z* 116.0706) is a discriminant molecule in *Tenebrio molitor*. This observation is in line with the EFSA opinion published in 2021 [5], which showed proline is the second-most abundant amino acid in *Tenebrio molitor* larvae.

In February 2022, the European Commission authorized the marketing of a third insect, *Acheta domesticus* (house cricket), as a food [29]. Together with *Tenebrio molitor* and locusts, these novel foods are permitted to be sold in frozen, dried, and powdered forms. However, there is a great need for high throughput and accurate methods capable of differentiating the insect species in dried and powdered forms. The Food and Agriculture Organization (FAO) has already highlighted possible food safety issues with edible insects, including pathogens, mycotoxins, pesticides, heavy metals, antimicrobials, and allergens [7]. The presence of these hazards is perhaps even more realistic and less controlled if fraudulent substitutions occur for profit. Note that it would be highly advantageous to be able to rapidly discriminate the insect species, thus circumventing the need for further time-consuming analyses associated with sequencing methods [30]. The DART-HRMS chemical profiles revealed clear interspecies differences that served as the basis for chemometric analysis and classifier build-up. The results show the great potential of DART-HRMS for the generation of unique species-specific chemical fingerprints that can be used for rapid identification of insect species. The performance of the random forest model was evaluated on a test set, resulting in a 100% overall accuracy. Further blind-controlled tests, with an independent batch of samples, are still necessary to establish the real and late-stage performances of this non-targeted method [31,32]. It is worth noticing that the chemical fingerprints could be affected by the rearing system, developmental stage, dietary interventions, and exposure to different bacterial strains used as dietary sources. In order to minimize these effects, the training set should be populated by a high number of different insects’ powders obtained from a variety of breeding systems [33]. The method, while discussed here in the context of discriminating four edible insect species, is equally applicable to other insect powders that are expected to be approved in the near future. By eliminating or minimizing the extensive use of hazardous chemicals (no toluene, no chloroform) and chromatographic solvents, the technique can be considered sustainable. This is the first application of DART-HRMS to the authentication of novel food.

## 5. Conclusions

The technique outlined here is the first demonstration of a rapid chemical fingerprint-based method for the identification of edible insect species in powder form. Future efforts will be directed at enlarging the reference set of the current non-targeted method, as well as developing a predictive model able to discriminate products obtained from other insect species. Moreover, further studies are necessary to authenticate the insect powders and detect their possible partial or total adulterations. Our results demonstrate the reliability of the DART-HRMS as a screening method in a future industrial scenario to detect complete substitution of insect powders along the farm to fork chain.

## Figures and Tables

**Table 1:** List of discriminant (±) DART-HRMS compounds that enable the identification of the four species of insects. The observed *m*/*z*, theoretical *m*/*z*, error (ppm), elemental formula, type of ion, and tentative assignment are listed.

Insect	Observed *m*/*z*	Theoretical *m*/*z*	Error (ppm)	Elemental Formula	Type of Ion	Tentative Assignment	References
*Tenebrio* *molitor*	114.0556	114.0561	−4.4	C_5_H_9_NO_2_	[M-H]^−^	proline	[5]
	116.0706	116.0706	0	C_5_H_9_NO_2_	[M+H]^+^	proline	[5]
	128.0351	128.0348	2.4	C_5_H_9_NO_4_	[M-H]^−^	glutamic acid/oxoproline	
	402.4300	402.4306	−1.5	C_25_H_52_O_2_	[M+H]^+^	erythro-6,8-pentacosanediol	
*Acheta* *domesticus*	106.0864	106.0863	0.9	C_4_H_8_O_2_	[M+NH4]^+^	FA C4:0 (butyric acid)	
	255.2325	255.2330	−1.9	C_16_H_32_O_2_	[M-H]^−^	FA C16:0 (palmitic acid)	[25]
	279.2328	279.2330	−0.7	C_18_H_32_O_2_	[M-H]^−^	FA C18:2 (linoleic acid)	[25]
*Bombyx* *mori*	117.0194	117.0193	0.9	C_4_H_6_O_4_	[M-H]^−^	succinic acid	
	191.0570	191.0561	−4.7	C_7_H_12_O_6_	[M-H]^−^	quinic acid	
	277.2176	277.2173	1.1	C_18_H_30_O_2_	[M-H]^−^	FA C18:3 (linolenic acid)	[26]
	279.2311	279.2319	−2.9	C_18_H_30_O_2_	[M+H]^+^	FA C18:3 (linolenic acid)	[26]
	296.2576	296.2584	−2.7	C_18_H_30_O_2_	[M+NH4]^+^	FA C18:3 (linolenic acid)	[26]
*Hermetia* *illucens*	89.0242	89.0244	−2.2	C_3_H_6_O_3_	[M-H]^−^	lactic acid	
	129.0193	129.0193	0	C_5_H_6_O_4_	[M-H]^−^	N/A	
	255.2325	255.2330	−1.9	C_16_H_32_O_2_	[M-H]^−^	FA C16:0 (palmitic acid)	[27,28]
	281.2483	281.2486	−1.1	C_18_H_34_O_2_	[M-H]^−^	FA C18:1 (oleic acid)	[27,28]
	439.3769	439.3788	−4.2	C_27_H_52_O_4_	[M-H_2_O+H]^+^	diacylglycerol DG(24:0)	

FA: fatty acid; DG: diacylglycerol.

**Table 2:** Statistical figures of merit (sensitivity, specificity, and accuracy) for the random forest model obtained in cross-validation on the training set and validation on the test set. Numbers of samples correctly classified are also reported.

Merged Dataset	Sensitivity	Specificity	Accuracy	Samples Correctly Classified
**Training set**	100%	100%	100%	25/25
**Test set**	100%	100%	100%	8/8

## Data Availability

Data can be enquired from atata@izsvenezie.it.

## Supplementary Materials

The following supporting information can be downloaded at: https://www.mdpi.com/article/10.3390/foods11152264/s1, Figure S1: PCA scores plot; Figure S2: Blank spectrum of the ethylacetate acquired by DART-HRMS in negative ion mode; Figure S3: Blank spectrum of the ethylacetate acquired by DART-HRMS in positive ion mode; Figure S4: Blank spectrum of the MeOH: H2O (80:20) solvent acquired by DART-HRMS in negative ion mode; Figure S5: Blank spectrum of the MeOH: H2O (80:20) solvent acquired by DART-HRMS in positive ion mode; Table S1: Confusion matrix with the results of the cross-validation of the random forest classifier on training set. Repetitions of the spectra are included; Table S2: Confusion matrix with the results of the test of the random forest classifier on withheld test set. Repetitions of the spectra are included.
