# Supplementary material for: Authentication of Edible Insects’ Powders by the Combination of DART-HRMS Signatures: The First Application of Ambient Mass Spectrometry to Screening of Novel Food
Source: Foods. 2022 Jul 29;11(15):2264. doi: 10.3390/foods11152264 (PMC9368114; doi:10.3390/foods11152264)
Supplement: Supplementary file 1 [file foods-11-02264-s001.zip › foods-1815032-supplementary.pdf]

Authentication of edible insects' powders by the combination of DART-HRMS signatures. The first application of ambient mass spectrometry to screening of novel food

A. Tata[1$], A. Massaro[1$], F. Marzoli[2], B. Miano[1], M. Bragolusi[1], R. Piro[1] and S. Belluco[2]

1. Istituto Zooprofilattico Sperimentale delle Venezie, Laboratorio di Chimica Sperimentale, Vicenza, Italy

2. Department of Food Safety, Istituto Zooprofilattico Sperimentale delle Venezie, Legnaro, Italy

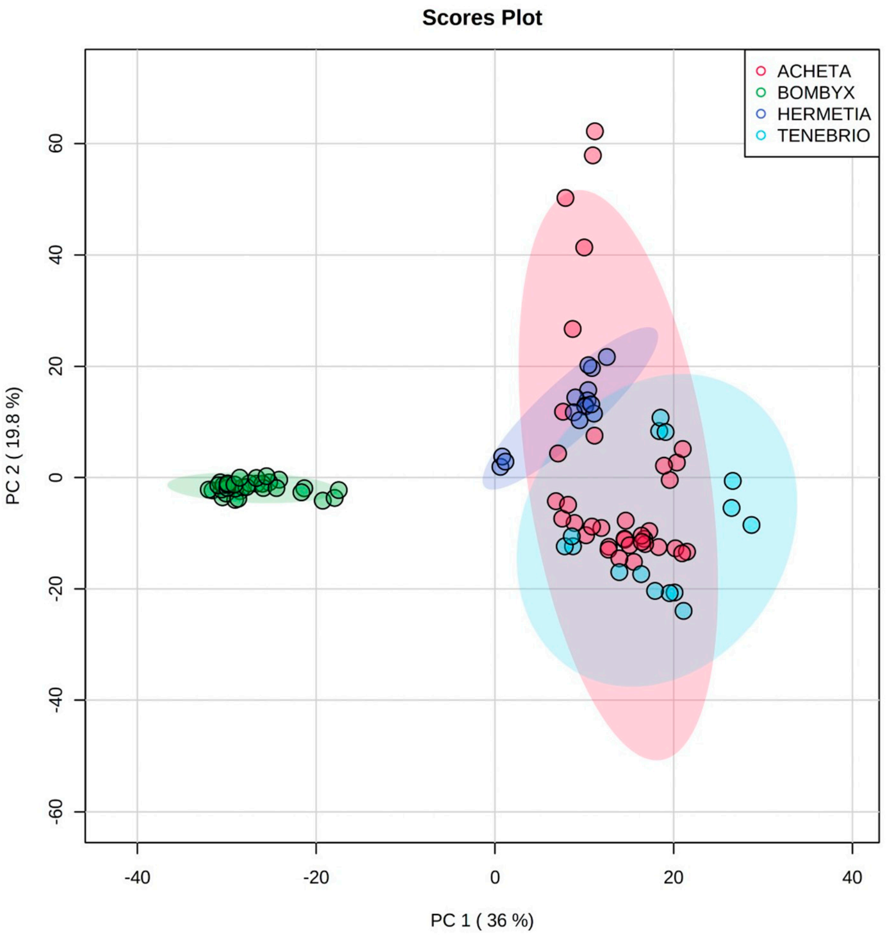

Figure S1. PCA scores plot.

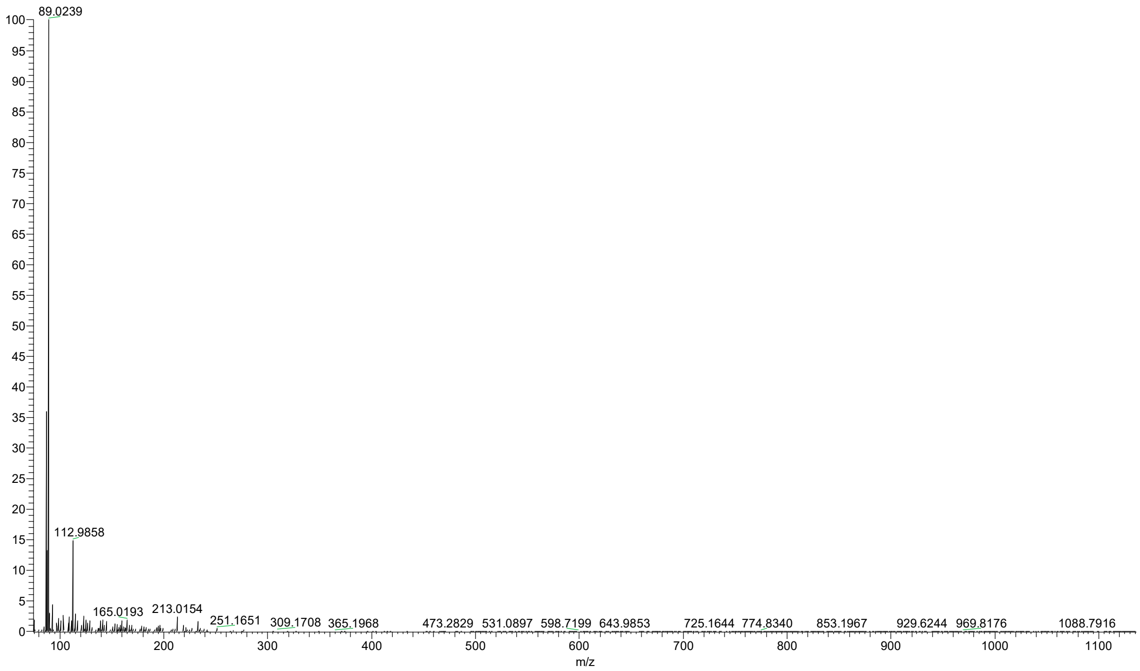

Figure S2. Blank spectrum of the ethylacetate acquired by DART-HRMS in negative ion mode.

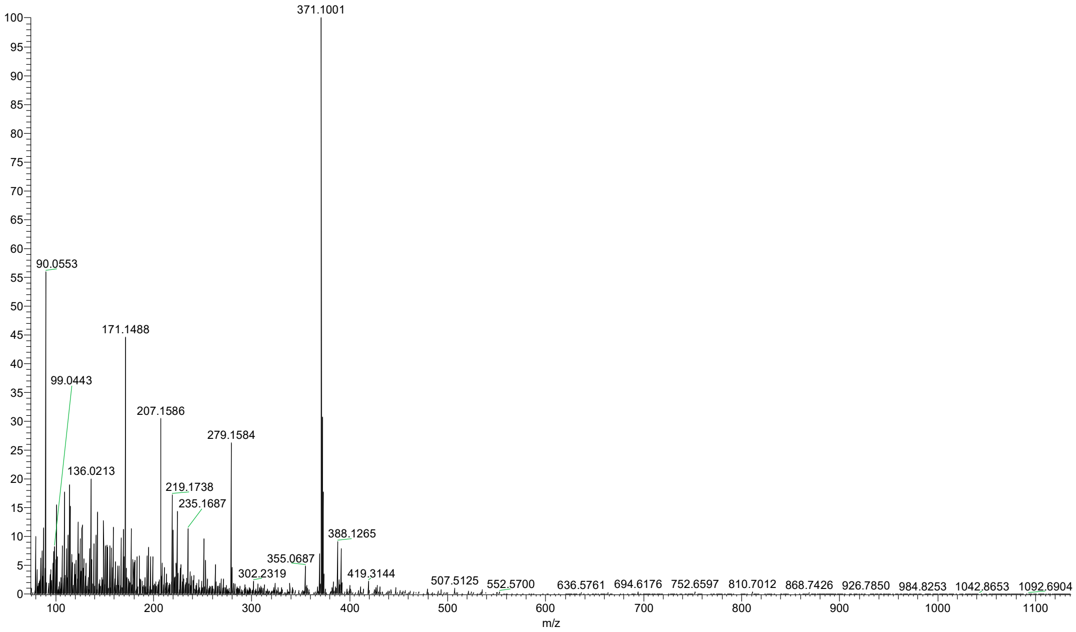

Figure S3. Blank spectrum of the ethylacetate acquired by DART-HRMS in positive ion mode.

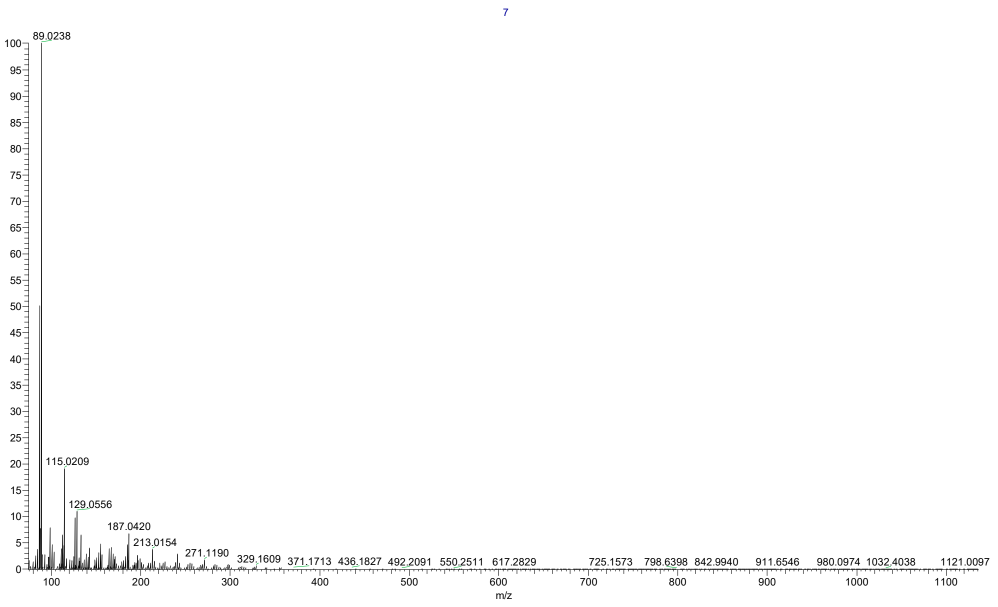

Figure S4. Blank spectrum of the MeOH: H_2O (80:20) solvent acquired by DART-HRMS in negative ion mode.

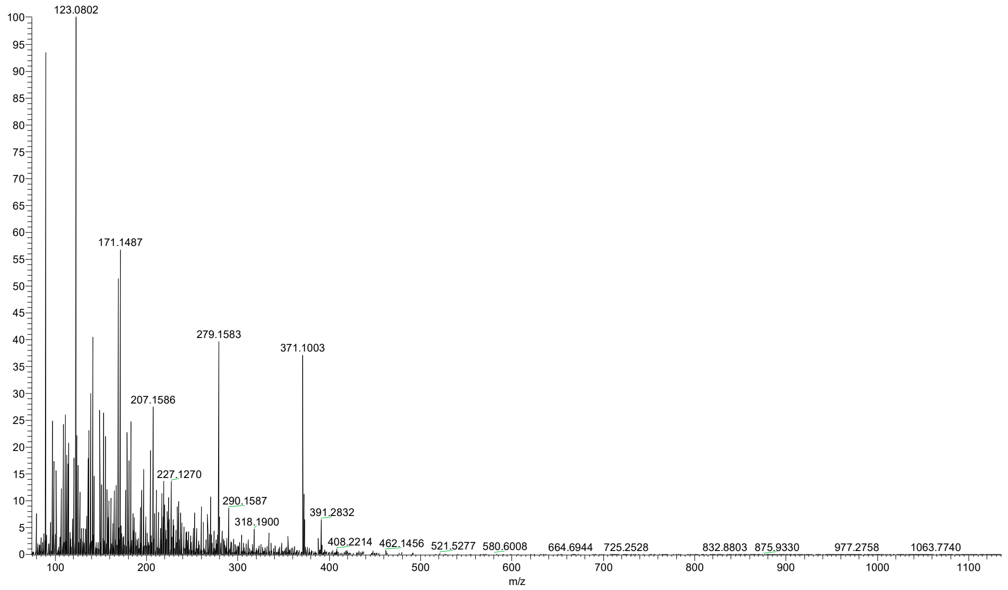

Figure S5 Blank spectrum of the MeOH: H_2O (80:20) solvent acquired by DART-HRMS in positive ion mode.

Table S1. **Confusion matrix with the results of the cross-validation of the random forest classifier on training set. Repetitions of the spectra are included.**

		Actual class			
		ACHETA	BOMBYX	HERMETIA	TENEBRIO
Predicted class	ACHETA	27	-	-	-
	BOMBYX	-	24	-	-
	HERMETIA	-	-	12	-
	TENEBRIO	-	-	-	12

Table S2. Confusion matrix with the results of the test of the random forest classifier on withheld test set. Repetitions of the spectra are included.

		Actual class			
		ACHETA	BOMBYX	HERMETIA	TENEBRIO
Predicted class	ACHETA	9	-	-	-
	BOMBYX	-	9	-	-
	HERMETIA	-	-	3	-
	TENEBRIO	-	-	-	3
